# Thoracic sympathetic block reduces respiratory system compliance

**DOI:** 10.1590/S1516-31802007000100003

**Published:** 2007-01-04

**Authors:** Fábio Ely Martins Benseñor, Joaquim Edson Vieira, José Otávio Costa Auler

**Keywords:** Epidural anesthesia, Autonomic nerve block, Respiratory mechanics, physiology, Lung compliance, Airway resistance, Anestesia epidural, Bloqueio nervoso autônomo, Mecânica respiratória, Complacência pulmonar, Resistência das vias respiratórias

## Abstract

**CONTEXT AND OBJECTIVE:**

Thoracic epidural anesthesia (TEA) following thoracic surgery presents known analgesic and respiratory benefits. However, intraoperative thoracic sympathetic block may trigger airway hyperreactivity. This study weighed up these beneficial and undesirable effects on intraoperative respiratory mechanics.

**DESIGN AND SETTING:**

Randomized, double-blind clinical study at a tertiary public hospital.

**METHODS:**

Nineteen patients scheduled for partial lung resection were distributed using a random number table into groups receiving active TEA (15 ml 0.5% bupivacaine, n = 9) or placebo (15 ml 0.9% saline, n = 10) solutions that also contained 1:200,000 epinephrine and 2 mg morphine. Under general anesthesia, flows and airway and esophageal pressures were recorded. Pressure-volume curves, lower inflection points (LIP), resistance and compliance at 10 ml/kg tidal volume were established for respiratory system, chest wall and lungs. Student's t test was performed, including confidence intervals (CI).

**RESULTS:**

Bupivacaine rose 5 ± 1 dermatomes upwards and 6 ± 1 downwards. LIP was higher in the bupivacaine group (6.2 ± 2.3 versus 3.6 ± 0.6 cmH_2_O, p = 0.016, CI = −3.4 to −1.8). Respiratory system and lung compliance were higher in the placebo group (respectively 73.3 ± 10.6 versus 51.9 ± 15.5, p = 0.003, CI = 19.1 to 23.7; 127.2 ± 31.7 versus 70.2 ± 23.1 ml/cmH_2_O, p < 0.001, CI = 61 to 53). Resistance and chest wall compliance showed no difference.

**CONCLUSION:**

TEA decreased respiratory system compliance by reducing its lung component. Resistance was unaffected. Under TEA, positive end-expiratory pressure and recruitment maneuvers are advisable.

## RESUMO

**CONTEXTO E OBJETIVO:**

Os benefícios pós-operatórios da anestesia peridural torácica (APT) na analgesia e respiração após toracotomias são conhecidos. Contudo, bloqueio simpático torácico pode desencadear hiperreatividade das vias aéreas. Este estudo pesou tais efeitos benéficos e indesejáveis na mecânica respiratória intra-operatória.

**TIPO DE ESTUDO E LOCAL:**

Estudo clínico, randomizado, duplo-cego realizado em hospital público terciário.

**MÉTODOS:**

Uma tabela de números aleatórios dividiu 19 pacientes submetidos a ressecção pulmonar parcial entre duas soluções administradas na APT: ativa (15 ml 0,5% bupivacaína, n = 9) ou placebo (15 ml 0,9% NaCl, n = 10). Ambas continham epinefrina 1:200,000 e morfina 2 mg. Sob anestesia geral, pressões esofágicas e de vias aéreas foram registradas. Curvas de pressão *versus* volume, pontos de inflexão inferior (PII), resistências e complacências sob volume corrente de 10 ml.kg^-1^ foram aferidos para sistema respiratório, parede torácica e pulmões. O teste t de Student foi realizado (p < 0,005), incluindo intervalos de confiança (IC).

**RESULTADOS:**

A dispersão cefálica e caudal da bupivacaína foi, respectivamente, de 5 ± 1 e de 6 ± 1 dermátomos. A curva PII foi maior no Grupo Bupivacaína (6,2 ± 2,3 *versus* 3,6 ± 0,6 cm H_2_O, p = 0,016, IC = −3,4 a −1,8). Complacências do sistema respiratório e pulmões foram maiores no Grupo Placebo (respectivamente 73.3 ± 10.6 *versus* 51.9 ± 15.5, p = 0,003, IC = 19,1 a 23,7, e 127,2 ± 31,7 *versus* 70,2 ± 23,1 ml.cm H_2_O^-1^, p < 0,001, IC = 61 a 53). Resistências e complacências da parede torácica não mostraram diferenças.

**CONCLUSÃO:**

APT diminui a complacência do sistema respiratório por reduzir seu componente pulmonar. Resistências não são afetadas. Sob APT, pressão positiva expiratória final e manobras de recrutamento são recomendáveis.

## INTRODUCTION

Adequate postoperative analgesia is considered to be a major key for reducing pul monary morbidity and improving the outcome.^1-3^

Thoracic epidural anesthesia (TEA) has been proposed as a reliable analgesic method following thoracic surgery.^3^ Most of these benefits, however, relate to the postoperative or to the so-called perioperative period. Some case reports have suggested that sympathetic block secon dary to thoracic or spinal anesthesia might cause impaired intraoperative ventilation by increasing airway resistance and bronchial reactivity.^4,5^

A similar controversy surrounds some aspects of respiratory mechanics in anesthetized humans. Although previous studies have demonstrated that systemic local anesthetic secondary to TEA does not alter airway resistance in humans, and even attenuates bronchial hyperreactivity,^6,7^ the effects of thoracic sympathetic block on respiratory mechanics, particularly on its compliance, have been poorly explained.

Pressure-volume curves are a feasible method for studying respiratory system mechanics, and the use of a low-flow inflation technique has been established as a reliable and quick method for obtaining these curves.^8,9^

The remaining doubts are whether the advantageous postoperative effects of TEA begin intraoperatively, and whether these benefits outweigh the undesirable intraoperative effects of thoracic sympathetic block.

## OBJECTIVE

To evaluate the effects of intraoperative thoracic sympathetic block on the elastic, viscoelastic and resistive properties of the respiratory system and its components (chest wall and pulmonary parenchyma), through analysis of pressure-volume curves obtained under quasi-static conditions.

## METHODS

### Patient selection

Patients requiring pulmonary segmental resection, pulmonary lobectomy, pulmonary biopsy or mediastinal nodular resection were eligible for the study. Those classified on the American Society of Anesthesiologists (ASA) scale as having a physical status score of 3 or higher were excluded, as were those presenting with moderate or severe obstructive respiratory disease, or any degree of restrictive respiratory disease, as diagnosed by spirometry and/or clinical signs. Written informed consent was obtained from each patient, and the study was approved by the hospital's Ethics Committee.

Patients were randomly assigned to one of two anesthetic solutions that were injected into the epidural space: 15 ml of 0.5% bupivacaine plus epinephrine 1:200,000 and 2 mg morphine chlorohydrate (bupivacaine group), or 15 ml 0.9% NaCl plus epinephrine 1:200,000 and 2 mg morphine chlorohydrate (placebo group). Randomization and blindness were achieved by having an anesthesiologist who was not involved in the study make the draw for the two solutions by means of a randomization table that identified the patients.

### Anesthesia

All patients were administered epidural and general anesthesia. They were premedicated with 10 mg of diazepam orally the night before surgery and 0.1 mg/kg of midazolam intramuscularly 45 minutes before surgery. Once in the operating room, the electrocardiogram (CB-5), arterial pressure, pulse oximetry and temperature were monitored.

Epidural anesthesia was performed in the T7-T8 interspace by the loss-of-resistance technique, with patients in the sitting position. If a loss of resistance could not be achieved in this interspace, attempts were made to use the T8-T9 and T9-T10 interspaces. Patients for whom locating the epidural space continued to be impossible after these three approaches were excluded from the study, in order to avoid a different spread of local anesthetic within the epidural space. Once the epidural space was located, the assigned solution was injected and an epidural catheter positioned in order to allow for postoperative analgesia. The patient was then maintained in the supine position for 30 mi nutes. Following this, an anesthesiologist who was not involved in the study and was unaware which solution had been injected tested the level of the block by applying a thermal bilateral stimulus to the mid-axillary and mid-clavicular lines, from the cervical region to the pubis. If there was a difference between the right and left sides, the lower anesthetic spread was recorded.

After the extent of TEA was assessed, the patient received 100% oxygen by means of a facemask for five minutes. Anesthesia was induced using 2 mg/kg of propofol and 0.5μg/kg of sufentanil citrate, and oral tracheal intubation was performed by using a 37 left endobronchial tube (Smith Industries Medical Systems Inc./Portex, Keene, New Hampshire, United States), facilitated by 0.1 mg/kg of vecuronium bromide. Correct tube positioning was checked by means of fiberoptic bronchos-copy. Anesthesia was maintained using continuous infusion pumps (ANNE^TM^ Anesthesia Infuser, Abbott Laboratories, Chicago, Illinois, United States) for propofol, vecuronium and sufentanil and, when necessary, small boluses of these same agents.

After induction, radial and right atrial pressures were invasively measured via intravascular catheters.

During these and the subsequent procedures, the patients were ventilated using a circulating system with CO_2_ absorber connected to the anesthesia machine (Intermed Linea anesthesia apparatus, São Paulo, Brazil) in volume controlled mode, square-wave (constant) flow of 30 l/min, respiratory rate of 10 breaths per minute, tidal volume of 8 ml per kilo and positive end-expiratory pressure (PEEP) level of 5 cmH_2_O. The fresh gas flow composition was a mixture of air and oxygen in equal parts. Inspired and expired gas analysis was performed with a Capnomac Ultima respiratory monitor (Datex Instrumentarium, Helsinki, Finland).

### Respiratory mechanics data acquisition

Data acquisition on respiratory mechanics was performed prior to the start of surgery. Immediately before each measurement, the airways were cleaned in order to remove accumulated mucus. Thus, ventilation was stopped, the fresh gas manifolds were closed, and the ventilator was adjusted as follows: volume-controlled mode, square-waveform (constant) flow of 6 l/min, tidal volume of 1000 ml, respiratory rate of three breaths per minute, and zero positive end-expiratory pressure (ZEEP). An inspiratory pause of five seconds was applied after this tidal volume was reached, in order to obtain a plateau and determine the resistance.

The resistance relative to the tracheal cannula was measured by connecting the proximal end of the cannula to the anesthesia machine Y-piece, with a pneumotachograph inserted between them and the distal end of the cannula left open, as described previously.^10^ This value was removed from the peak airway pressure before analysis.

Total resistance (Rmax), minimum resistance (Rmin) and additional resistance (DR) were determined for the respiratory system, chest wall and lungs using previously described methods.^7,11-13^ Rmin reflects the opposition to air flow through the airways in the respiratory system (Rmin, rs), chest wall (Rmin, w) and lung parenchyma (Rmin, L). DR represents the additional resistance secondary to volume redistribution and/or tissue relaxation following airway flow cessation in the respiratory system (DR, rs), chest wall (DR, w) and lung parenchyma (DR, L).^12^

Airway pressure (Paw) and inspiratory and expiratory flows were measured using a variable-area pneumotachograph (Bicore CP-100 respiratory monitor, Irvine, California, United States). The sensor (Var-Flex^®^ Flow Transducer, Allied Healthcare, California, United States) was inserted between the proximal tip of the endobronchial tube and the Y-piece. For each patient, the anesthesia apparatus flow controls were calibrated by means of a Timeter RT-200 (Allied Healthcare, California, United States) to ensure that the set flows were absolutely correct during measurements.

Esophageal pressure was measured using an air-filled catheter (SmartCath^®^ Esophageal Catheter, BEAR Medical Systems, California, United States) inserted orally and connected to the Bicore CP-100 monitor. Catheter positioning in the lower third of the esophagus was confirmed by means of the occlusion test.^14^

Tidal volumes were obtained by integration of the flow curve.

### Data formatting and analysis

The analog Bicore signals were recorded in ASCII format on a PC (IBM Computers, São Paulo, Brazil) by using an analog-to-digital converter (CAD 12 bit/32 channels, Lynx, São Paulo, Brazil) for one minute at 200 Hz. The files were converted to Excel for Windows 2000 format (Microsoft, São Paulo, Brazil) before analysis. Analysis of the flow curve allowed determination of the beginnings of the inspiratory and expiratory phases, as well as the beginning and end of the inspiratory pause, in accordance with a previous study.^10^ The flow values were double-checked by observing the inspiratory time on the pressure curve (the 1000 ml tidal volume had to be reached in exactly 10 seconds to assure a flow equal to 100 ml/s).

Intrinsic PEEP (PEEPi), which was considered to be any pressure measured at zero flow, was subtracted when detected during an expiratory pause of five seconds. After the pressure-volume curves were built, a polynomial trend line was obtained for each curve, to remove artifacts from the cardiac rhythm. These trend lines and the equations originating from them were used for the data analysis.

Quasi-static compliance for the respiratory system (Crs), chest wall (Cw), and lung parenchyma (CL) were calculated by dividing the tidal volume at end-inspiration by airway pressure (Paw), esophageal pressure (Pes) and the difference between them (Paw-Pes). The tidal volume used for statistical analysis was 10 ml/kg, as proposed by Gattinoni et al.^15^

The lower inflection point (LIP) was obtained by finding the intersect between the starting compliance (the ratio between the first 100 ml inflation and the corresponding pressure) and the inflation compliance (the slope of the pressure-volume curve in its most linear segment), also in accordance with the method proposed by Gattinoni et al.^15^

### Statistical analysis

Statistical analysis was performed using the SAS software version 8.0 (SAS, São Paulo, Brazil). Patients’ characteristics were compared using Student's t test, except for the epidural puncture level and block spread, which were compared using the Mann-Whitney Rank Sum Test. Fisher's Exact test was used to compare the gender variable. Resistance and compliance values for respiratory system, chest wall and lungs were compared using Student's t test. Pflex was compa red using the Mann-Whitney Rank Sum Test, since its analysis did not pass the normality test. Values of 0.05 or lower were considered significant. Confidence intervals (CI) were calculated for differences between mean values and adjusted to the sample size.

## RESULTS

A total of 29 patients were initially recruited. Of these, four were excluded because of uncorrected flow settings that were noticed during the analysis; one because epidural catheter placement was impossible; two because esophageal catheter location in accordance with the established reference method^14^ was impossible; two because the plateau interval was shorter than five seconds; and one that was considered to be an epidural block failure since no spread of sensory block could be detected even though this patient received the bupiva-caine solution. Consequently, the compliance and resistance of 19 patients (9 in the bupiva-caine group and 10 in the placebo group) were analyzed. The demographic characteristics and spreads of the sensory epidural block for these 19 patients are presented in Table 1.

**Table 1.** Characteristics and ventilation settings of the 19 patients studied for respiratory mechanics, presented as mean ± standard deviation

	0.9% saline	0.5% bupivacaine	p [CI]
Age (years)	46.4 ± 15.4	43.1 ± 13.4	0.628
Gender (male/female)	5/5	1/8	0.141
Weight (kg)	64.6 ± 7.7	68.8 ± 9.3	0.298
Height (m)	1.64 ± 0.1	1.61 ± 0.1	0.324
Body mass index (kg/m^2^)	24.2 ± 3.5	26.6 ± 3.5	0.147
Puncture level*	7	8	0.485
Cranial spread*	0	5 ± 1	< 0.001
Caudal spread*	0	6 ± 1	< 0.001
Tidal volume (TD, ml)	591.4 ± 63.6	598.3 ± 77.3	0.849
FIO2 (mmHg)	0.68 ± 0.1	0.68 ± 0.2	0.981
SpO_2_ (mmHg)	99 ± 1	99 ± 1	0.412
EtCO2 (mmHg)	41 ± 4	36 ± 4	0.022 [4.5 to 5]
PaO_2_ (mmHg)	327.9 ± 104	264.8 ± 138	0.273 [47.2 to 79.1]
PaCO_2_ (mmHg)	42.2 ± 5.3	40.8 ± 3.7	0.494 [2.2 to 0.8]

* Height of block presented as number of dermatomes.

The patients underwent spirometry evaluation one day before surgery, in order to apply the exclusion criteria. Those scheduled for the bupivacaine group presented mean forced vital capacity of 97.8% ± 7.9% of the predicted, forced expiratory volume in one second (FEV1) of 96.9% ± 7.3% of the predicted and a mean forced expiratory flow rate measured over the middle portion of the forced vital capacity (FEF_25-75_) of 87.8% ± 9.1% of the predicted. The patients from the placebo group presented mean forced vital capacity of 98.1% ± 17.8%, FEV1 of 88.1% ± 20.2% and mean FEF_25-75_ of 85.1% ± 23.7%. The measured spirometric values in both groups were within normal ranges for the Brazilian population.^16^ Although the smoking habit was not an exclusion criterion, only five patients (three in the bupivacaine group and two in the placebo group) had smoked over the last two years before this study. Among these, there was one active smoker in each group at the time of the study.

The lower inflection points were higher in the bupivacaine group than in the placebo group (6.2 ± 2.3 and 3.6 ± 0.6 cmH_2_O respectively, p = 0.016, CI = −3.4 to −1.8).

**Figure 1:**
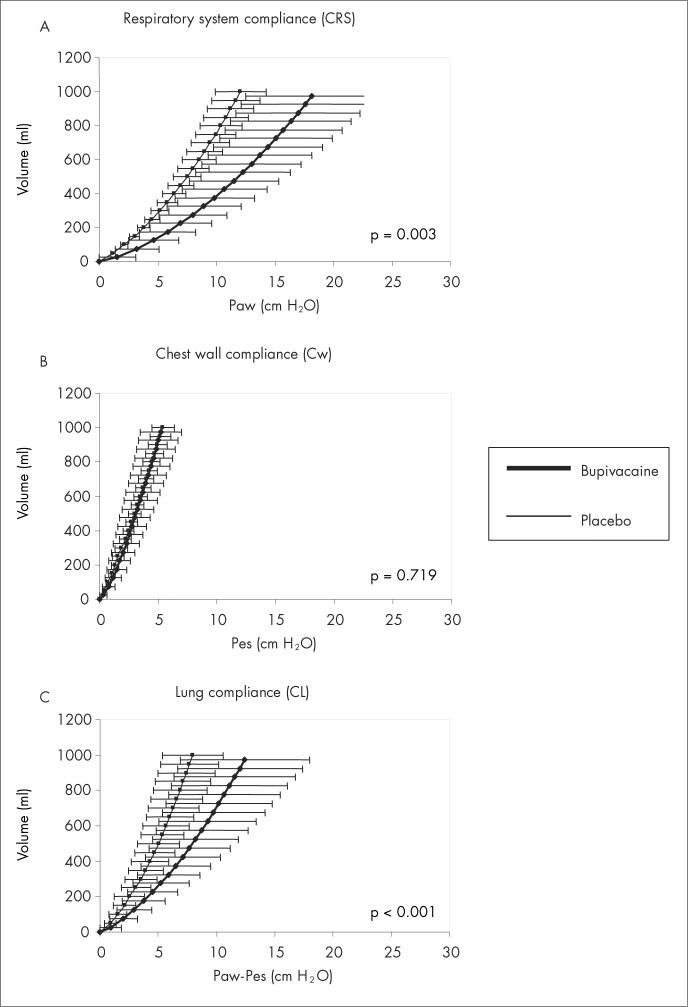
Comparative results between bupivacaine and placebo groups showing mean ± standard deviation (SD) compliance curves for respiratory system (A), chest wall (B) and lung parenchyma (C), and also the statistical differences found (P). Paw = airway pressure; Pes = esophageal pressure.

Higher respiratory system compliance was observed among patients assigned to the placebo than among those assigned to the bupivacaine solution, for a tidal volume of 10 ml/kg (73.3 ± 10.6 and 51.9 ± 15.5 ml/cmH_2_O respectively for placebo and bupivacaine solutions, p = 0.003, CI = 19.1 to 23.7). Lung compliance was also higher in the placebo group (127.2 ± 31.7 and 70.2 ± 23.1 ml/cmH_2_O, p < 0.001, CI = 61 to 53). No difference was found between the groups concerning chest wall compliance (186.3 ± 52.7 and 179.1 ± 30.7 ml/cmH_2_O for the bupivacaine and placebo groups, respectively, p = 0.719, CI = −17.5 to 3.1). Respiratory system, chest wall and lung compliance curves are presented respectively in Figures 1A, B and C.

There was no difference between the two groups regarding the resistance of the respiratory system or its lung and chest wall components. The calculated resistance is presented in Table 2.

**Table 2.** Airway pressures and resistance in the 19 patients studied

	0.9% saline	0.5% bupivacaine	p [CI]
P'max, aw	12.3 ± 2.1	18.9 ± 5.6	0.003 [-8.3 to −5]
P2, aw	10.9 ± 2.4	17.1 ± 5.2	0.003 [-7.5 to −4.9]
P1, aw	11.9 ± 2.2	18.3 ± 5.3	0.009 [-7.5 to −4.9]
Pmax, es	5.3 ± 1.1	4.9 ± 2.1	0.565 [0 to 0]
P2, es	4.5 ± 1.3	4.2 ± 1.9	0.696 [0 to 1]
Rmax, rs	13.8 ± 5	18.1 ± 8.4	0.192 [-5.9 to −2.7]
Rmax, w	8.1 ± 4.7	6.6 ± 3.7	0.443 [2 to 1.1]
Rmax, L	5.7 ± 2.7	11.5 ± 8.7	0.059 [-8.6 to −3]
Rmin, rs	3.8 ± 2	6.1 ± 5.6	0.223 [-4 to 0]
Rmin, L	3.8 ± 2	6.1 ± 5.6	0.223 [-4 to −0.7]
DR, rs	10.1 ± 3.9	12 ± 4.6	0.349 [-2.2 to −1.6]
DR, w	8.1 ± 4.7	6.6 ± 3.7	0.443 [2 to 1.1]
DR, L	2 ± 1.5	5.5 ± 5.4	0.061 [-5.4 to −1.7]

Calculated resistance presented as mean ± standard deviation. P'max, aw = maximum tracheal pressure minus pressure generated by the tracheal cannula (cmH_2_O); P2, aw = pressure at end of slow decay to a inspiratory airway plateau (cmH_2_O); P1, aw = pressure at the end of fast drop during the airway inspiratory pause (cmH_2_O); Pmax, es = maximum esophageal pressure (cmH_2_O); P2, es = esophageal pressure at end of inspiratory pause (cmH_2_O); Rmax = total resistance (cmH_*2*_O.s.L^-1^) generated by airway (Rmax, rs), chest wall (Rmax, w) and lung parenchyma (Rmax, L); Rmin = true airway (Rmin, rs) and lung (Rmin, L) resistance (cmH_2_O.s.L^-1^); and DR = residual resistance (cmH_2_O.s.L^-1^) of respiratory system (DR, rs), chest wall (DR, w) and lung parenchyma (DR, L). CI = Confidence Index.

## DISCUSSION

Thoracic sympathetic block secondary to thoracic epidural anesthesia was associated with decreased compliance of the respiratory system and its lung component, in patients undergoing mechanical ventilation. The chest wall component and the resistance of the respiratory system did not present any influence from the epidural block.

Several mechanisms can lower lung compliance, including atelectasis, increased smooth muscle tone and stimulation of other contractile elements in the airways or lung parenchyma and small airway closure.

Computed tomography has shown that pulmonary atelectasis is a common finding following the induction of anesthesia, occurring in almost 90% of all anesthetized patients.^17,18^ Atelectasis during anesthesia can be formed by reduced transmural alveolar distending pressure (compression atelectasis), gas absorption when using high-oxygen air mixtures (absorption atelectasis) or reduced surfactant production or action.^19,20^ The formation of atelectasis right after induction and the use of similar air mixtures in both groups suggest compression atelectasis as the probable etiology. There is no previous information suggesting a synergistic effect of general and thoracic epidural anesthesia on atelectasis formation, but there is a possibility that atelectasis after muscle paralysis, as demonstrated by Tokics et al.,^21^ may be further increased under epidural anesthesia. As the risks imposed by pulmonary artery catheterization were not justifiable in most of the patients studied, respiratory shunting was not calculated. Nevertheless, the finding of similar PaCO_2_ values in both groups and of reduced EtCO_2_ in the bupivacaine group suggests a ventilation-to-perfusion mismatch, probably secondary to the respiratory component, since there was no documented shift in the distribution of intrathoracic blood volume or pulmonary blood volume during epidural anesthesia.^22^

Increased smooth muscle tone or stimulation of other contractile elements in the airways induced by TEA should be associated with increased airway resistance. Although the values of respiratory system resistance (R, rs and its components) and interrupter lung resistance (Rmin, L) found in both groups were significantly higher than the corresponding values previously reported in normal anesthetically paralyzed humans,^23^ no significantly higher values were found in the bupivacaine group. Previous reports on the effect of TEA on respiratory system resistance in patients with documented bronchial hyperreacti vity showed increased acetylcholine threshold concentration, but this was correlated with local anesthetic blood serum concentration rather than with any effects from epidural sympathetic blockade.^6^ These results suggest that pulmonary sympathetic innervation effects on airway resistance are not relevant for clinical practice.

Small airway closure, either as a result of higher tonus in small airways or as a result of reduced FRC, would be a possible mechanism accounting for the diminished CL and the significantly higher LIP in the bupivacaine group. The slightly, although not statistically significant, increased residual resistance in the bupivacaine group, either from the lung tissue or from the small airways, may have contributed towards a difference that could be noticed as reduced compliance rather than as enhanced resistance of the respiratory system. The contracted peripheral airway may stretch the lung tissue, thus decreasing its compliance, as showed by the rightward shift of the pressure-volume curve in the bupivacaine group (Figure 1). It is also possible that sympathetic blockage may trigger isotonic contraction of lung tissue. Peripheral lung tissue has been identified as having the ability to respond directly to contractile stimulation, thus suggesting that lung parenchyma might play a role in obstructive diseases.^24^ It seems reasonable to consider the small airways as a possible site for sympathetic direct action.

This study was not designed to investigate the intraoperative effects of TEA, but those relating to the sympathetic blockade that is secondary to it. Thoracic epidural anesthesia was the tool that made thoracic sympathectomy possible. This is the reason why epinephrine was added to both solutions. We did not intend to evaluate whether the findings detected might be related to the sympathetic blockade or to the action of bupivacaine itself.

It would be difficult to ascribe the results to the gender composition of the bupivacaine and placebo groups, since the medical literature does not establish differences for respiratory mecha nics between male or female subjects, to the best of our knowledge. It is also important to emphasize that we studied patients with preserved respiratory function, as confirmed by the preoperative spirometric and laboratory analyses.

An advantageous correlation between postoperative analgesia, particularly with TEA, and better postoperative respiratory function has already been established. Ease of chest expansion, ability to cough and cooperation with physiotherapy work assure less atelectasis, pulmonary infection and respiratory failure. This study intended to evaluate whether such respiratory improvement begins intraoperatively or whether the postoperative benefits can hide intraoperative drawbacks. On reducing lung compliance, probably by increasing atelectasis, it may be assumed that intraoperative thoracic sympathetic block increases the postoperative work of breathing and consequently makes weaning more difficult. Besides atelectasis, another possibility is that decreased lung compliance is a consequence of small airway closure and reopening during tidal breathing, thus implying a risk of low lung volume injury. Both possibilities must be considered separately, since they are unrelated.^25^ Tomographic studies may be useful for clarifying this question. In both hypotheses, however, undesirable effects could be minimized by applying adequate PEEP levels.

## CONCLUSIONS

Intraoperative thoracic sympathetic block secondary to epidural anesthesia with bupivacaine reduces lung compliance. This effect seems to have no clinical relevance in healthy patients, but may become important for those presenting respiratory diseases before anesthesia. Since atelectasis was the most probable source of reduced compliance in this study, the use of adequate PEEP values associated with lung recruitment maneuvers and low fractions of oxygen in inspired gas may be recommended when performing intraoperative thoracic sympathetic block associated with general anesthesia.
